# Supplementary material for: Yersinia enterocolitica-Derived Outer Membrane Vesicles Inhibit Initial Stage of Biofilm Formation
Source: Microorganisms. 2022 Nov 29;10(12):2357. doi: 10.3390/microorganisms10122357 (PMC9786825; doi:10.3390/microorganisms10122357)
Supplement: Supplementary file 1 [file microorganisms-10-02357-s001.zip › microorganisms-1925087-supplementary.pdf]

Supplementary Materials

Supplementary figures

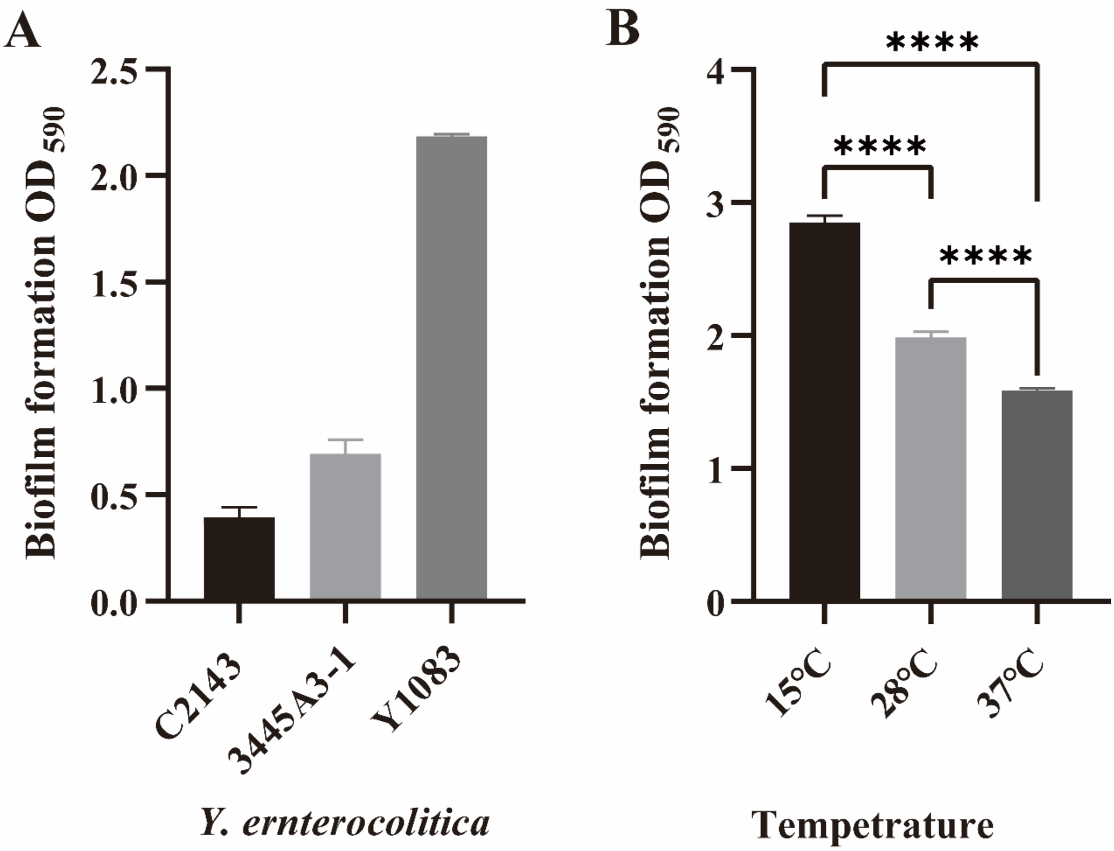

Supplementary Figure S1. Biofilm formed by *Y. enterocolitica*. (A) *Y. enterocolitica* strains Y1083, 3445A3-1 and C2143 were cultured at 28°C for 48 h. (B) Strain Y1083 was cultured at 15°C, 28°C and 37°C for 48 h, respectively. Biofilm formation was measured by crystal violet staining. One-way ANOVA was used followed by Tukey's multiple-comparison test using GraphPad Prism version 8.0.1 to assess significance. Error bar indicate the standard deviations of seven measurements. ****, $p<0.0001$.

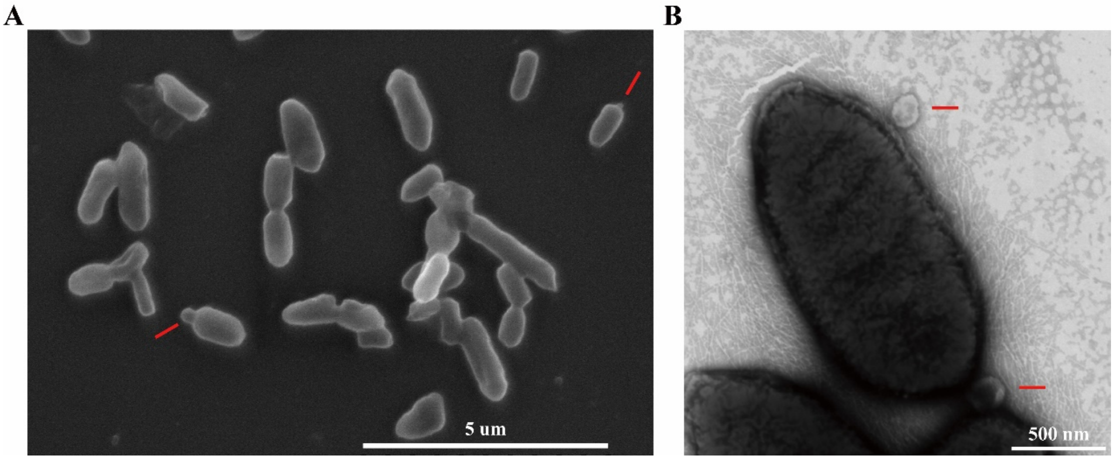

Supplementary Figure S2. *Y. enterocolitica* Y1083 observed by SEM and TEM. (A) SEM was used to observe *Y. enterocolitica* Y1083. (B) TEM was used to observe *Y. enterocolitica* Y1083. Red arrows point to broken OMVs.

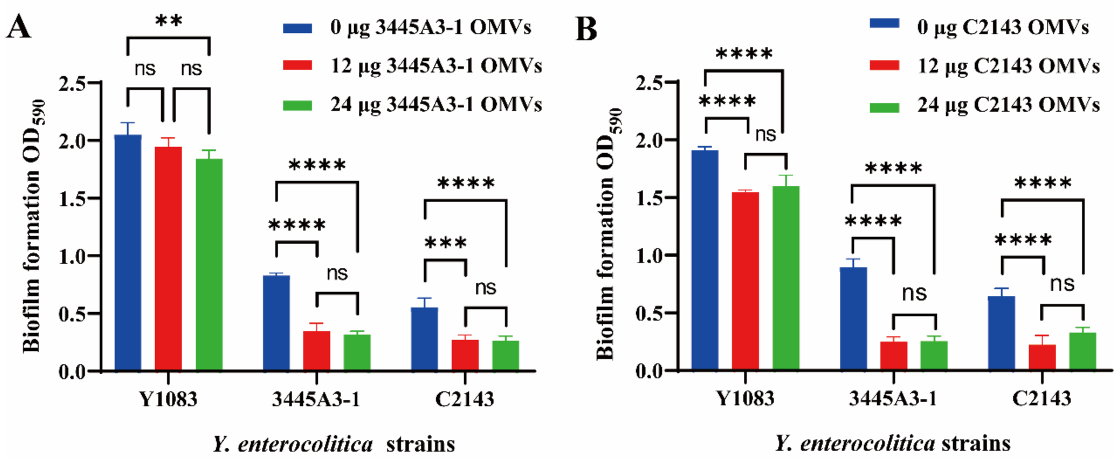

Supplementary Figure S3. The OMVs of *Y. enterocolitica* strains 3445A3-1 and C2143 inhibit biofilm formation. (A). (B) One-way ANOVA was used followed by Tukey's multiple-comparison test using GraphPad Prism version 8.0.1 to assess significance. Error bar indicate the standard deviations of seven measurements. ns, p>0.05, **, p<0.005, ***, p<0.0005, ****, p<0.0001.

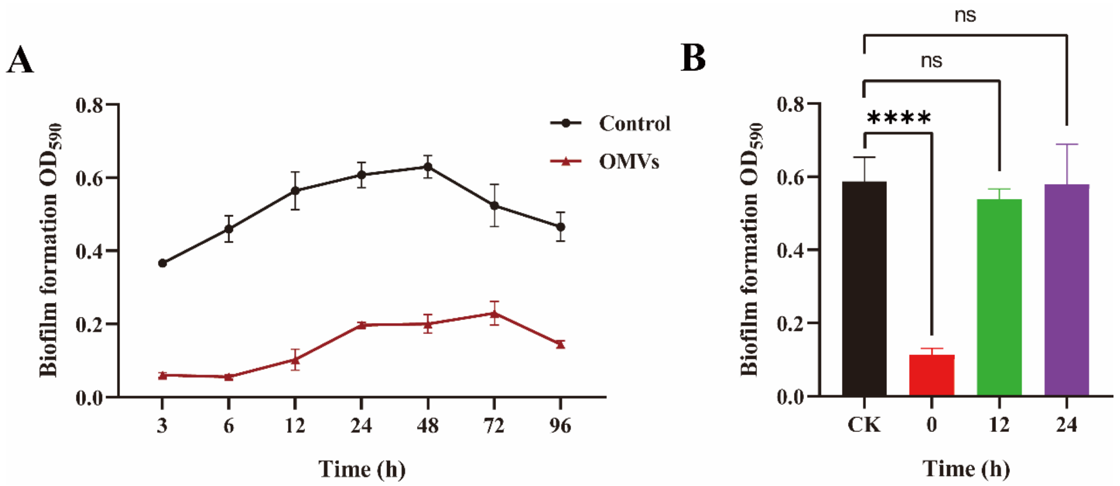

Supplementary Figure S4. The effect of OMVs to inhibit biofilm formation. (A) After adding 12 μg OMVs and *Y. enterocolitica* 3445A3-1 for 3, 6, 9, 12, 24, 48, 72 and 96 h, the amount of biofilm formation was measured by crystal violet staining. (B) When *Y. enterocolitica* 3445A3-1 grown 0, 12 and 24 h, 12 μg OMVs was added, respectively. One-way ANOVA was used followed by Tukey's multiple-comparison test using GraphPad Prism version 8.0.1 to assess significance. Error bar indicate the standard deviations of seven measurements. ns, p>0.05, ****, p<0.0001.

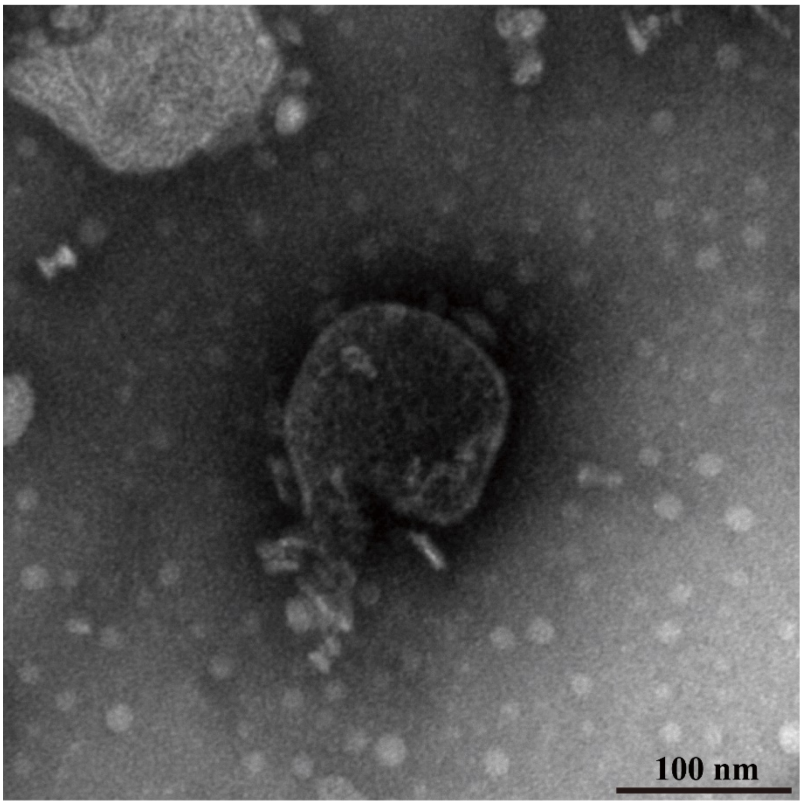

Supplementary Figure S5. TEM observe broken OMVs. OMVs was lysed by ultrasonic. TEM was used to observe the morphology of broken OMVs.

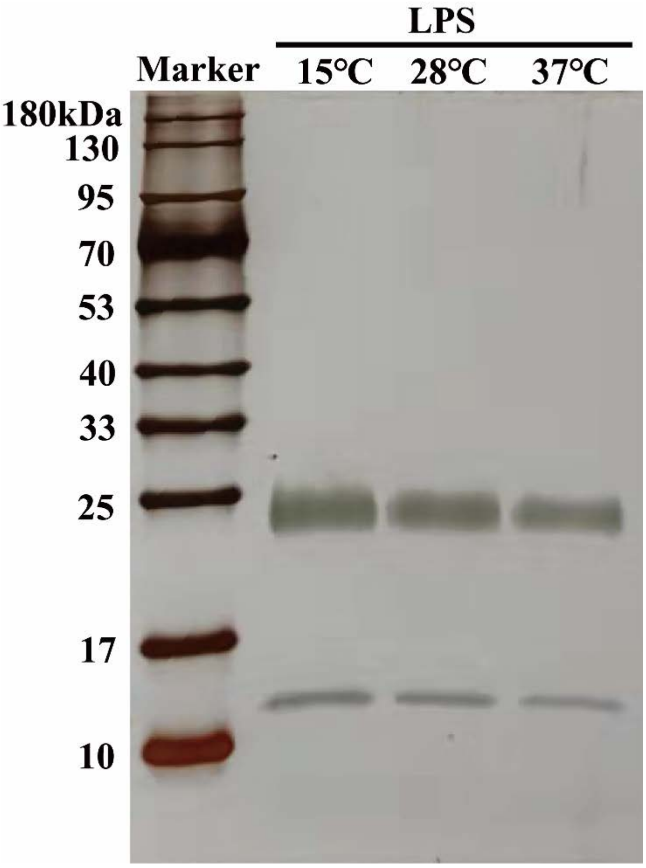

Supplementary Figure S6. LPS was analyzed by SDS-PAGE. LPS of OMVs were observed by SDS-PAGE separations. The protein gel was treated by silver staining.

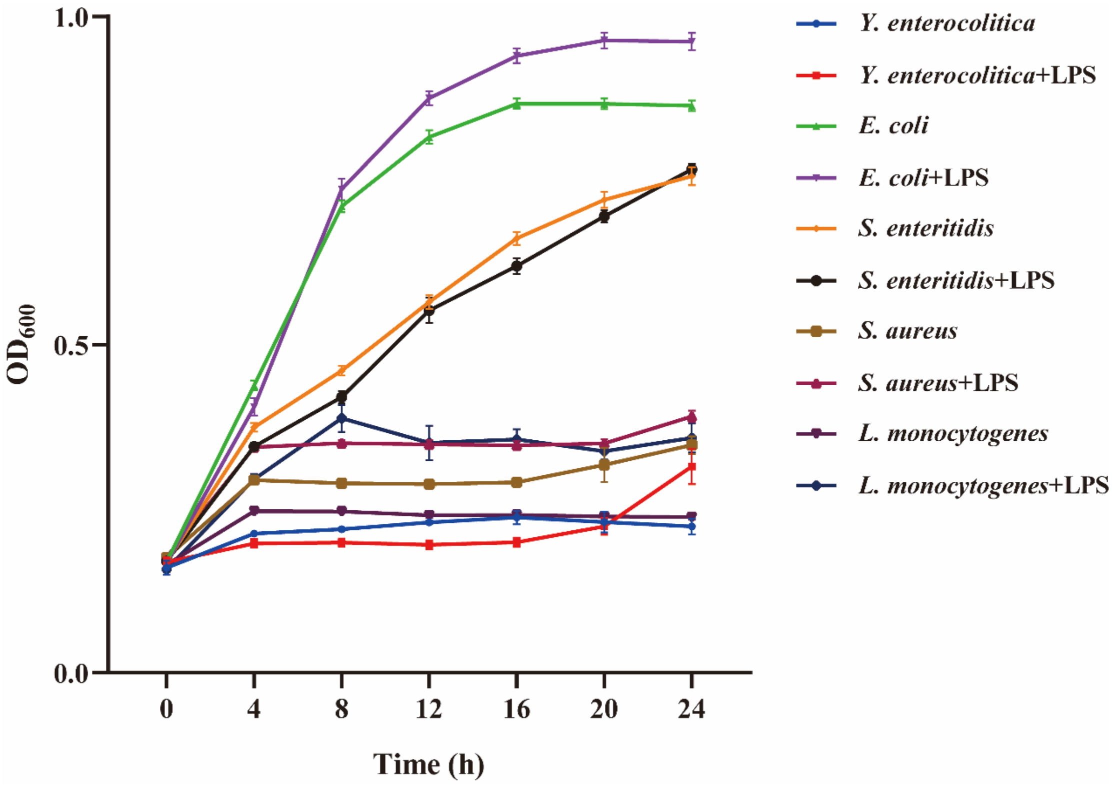

Supplementary Figure S7. Effects of LPS on growth curve of various bacteria. LPS was co-cultured with *Y. enterocolitica*, *E. coli*, *S. enteritidis*, *S. aureus* and *L. monocytogenes* at 37 for 24 h, respectively.
